# Researches on the Influence on the Gastric Juice by Adsorbents

**Published:** 1913-08

**Authors:** 


Researches on the Influence on the Gastric Juice by Adsorbents. Inaugural Dissertation by Fr. Wilhelm Greef of

Gottingen, 1911. Adsorbents are colloid, amorphous, substances with great surface area, adsorption consisting in the fixation of gaseous or dissolved substances without complete, homogeneous penetration of the molecule, which latter constitutes absorption. The principal adsorbents in common use are the various kinds of charcoal, kaolin, bismuth, iron hydroxid and neutralon. In a bibliography going back to 1834, he reviews the more or less empiric demonstration of the value of various adsorbents in diarrhoea, dysentery, etc. (fixation of bacterial toxins), arsenic and various alkaloid poisonings, in removing coloring matters in the sugar industry, organic analysis, etc.

He discusses various investigations into adsorption, including phenomena of surface tension. The less the concentration of a substance in solution, the more complete its adsorption. For acids, the nature of the adsorbent has very little influence, the fixation varying inversely as the concentration and directly with the molecular weight, at least for organic acids. Michaelis and Ehrenreich demonstrated that a marked difference in electric potential favors adsorption and that this difference diminishes during the process. Kaolin, for example, is negative, iron hydroxid positive. Thus kaolin adsorbs only such dyes as contain basic and amphoteric acid radicles and, hence, does not act at all on eosin, which is easily adsorbed by iron hydroxid (Cf. its action as a blood stain).

Quantitative estimates of HCl are obviously more accurate than of pepsin. In order to test the action of adsorbents on HCl, solutions were prepared of half, 1/5, 1/10, 1/20, 1/50 and 1/100 normal strength. Obviously, if each of these is titrated against the corresponding fractional alkaline solution (Na OH) any given quantity, for convenience lo c.c., will be neutralized by an equal quantity of the latter (Methyl red used as indicator). On adding the adsorbent, it was found, according to the general principle that adsorption varies inversely as the degree of concentration in solutions (though the word *inversely* is not applicable in a mathematic sense), that the amount of alkaline solution required for neutralization of the reminder of the HCl in solution, diminished proportionately, as the solutions used decreased in strength. Of course, if no adsorption had taken place, lo c.c. of any given strength would be required to neutralize acid solutions of the same amount of any corresponding strength. (It should also be noted that in each group of experiments, the same absolute quantity of adsorbent per lo c.c. of acid solution was used though it is implied that enough was used in each case to secure the maximum adsorbent effect).

